# Supplementary material for: Transposable element insertions shape gene regulation and melanin production in a fungal pathogen of wheat
Source: BMC Biol. 2018 Jul 16;16:78. doi: 10.1186/s12915-018-0543-2 (PMC6047131; doi:10.1186/s12915-018-0543-2)
Supplement: Supplementary file 1 — 3D7 accumulates more melanin than 3D1. Means and standard errors of gray values (0 = black, 255 = white) of at least 60 colonies of Z. tritici strains 3D1 and 3D7 at 7–12 days post inoculation (dpi). The experiment was performed three times with similar results. Asterisks indicate significant differences between 3D1 and 3D7 at each time point according to Kruskal-Wallis test (p values ≤ 0.05). n = number of colonies analyzed. (PDF 110 kb) [file 12915_2018_543_MOESM1_ESM.pdf]

Additional file 1. 3D7 accumulates more melanin than 3D1. Means and standard errors of gray values (0 = black, 255 = white) of at least 60 colonies of *Z. tritici* strains 3D1 and 3D7 at 7 - 12 days post inoculation (dpi). The experiment was performed three times with similar results. Asterisks indicate significant differences between 3D1 and 3D7 at each time point according to Kruskal-Wallis test (p-values ≤ 0.05). n = number of colonies analyzed.

Strains	**3D1** (n=64)		**3D7** (n=70)		**p-value (Kruskal-Wallis)**
dpi	Mean Gray Value	Standard error	Mean Gray Value	Standard error	
7	133	1.7	87	2.4	< 2.2e-16*
8	110	2.5	65	2.2	< 2.2e-16*
9	80	1.7	63	1.0	6.7e-14*
10	69	1.2	58	1.6	6.2e-12*
11	64	1.1	57	3.7	0.14
12	46	1.0	50	1.3	0.05
