# Supplementary material for: Transposable element insertions shape gene regulation and melanin production in a fungal pathogen of wheat
Source: BMC Biol. 2018 Jul 16;16:78. doi: 10.1186/s12915-018-0543-2 (PMC6047131; doi:10.1186/s12915-018-0543-2)
Supplement: Supplementary file 2 — Comparison of the QTLs obtained using the genome of the reference strain IPO323 and of the parental strain 3D7. (PDF 32 kb) [file 12915_2018_543_MOESM2_ESM.pdf]

Additional file 2. Comparison of the QTLs obtained using the genome of the reference strain IPO323 and of the parental strain 3D7.

	**QTL mapped to IPO323**	**QTL mapped to 3D7**
Confidence Interval (95%)	43429 bp (in IPO323)	18135 bp (in 3D7)
LOD score at peak (8 dpi, control)	32.2	29.95
p-value	<0.001	<0.001
Number of genes in the confidence interval	12	6
QTL peak	intergenic region before *Zt09_11_00187*	49 bp downstream of *Zt09_11_00190*
Peaking marker	581446 bp	679659 bp
Confidence interval (start - end)	559732 bp - 603161 bp (in IPO323)	661524 bp - 679659 bp (in 3D7)
Number of markers used for mapping on chromosome 11	252	2234
