# Supplementary material for: Transposable element insertions shape gene regulation and melanin production in a fungal pathogen of wheat
Source: BMC Biol. 2018 Jul 16;16:78. doi: 10.1186/s12915-018-0543-2 (PMC6047131; doi:10.1186/s12915-018-0543-2)
Supplement: Supplementary file 3 — Genes in the 95% confidence interval of the QTL obtained using the genome of the parental strain 3D7. Genes in the Pks1 gene cluster are indicated in bold. The total number of synonymous (Syn) and non-synonymous (Non-Syn) SNPs between both parental strains is indicated. Insertions upstream of the coding sequence are also indicated. (PDF 35 kb) [file 12915_2018_543_MOESM3_ESM.pdf]

Additional file 3. Genes in the 95% confidence interval of the QTL obtained using the genome of the parental strain 3D7. Genes in the *Pks1* gene cluster are indicated in bold. The total number of synonymous (Syn) and non-synonymous (Non-Syn) SNPs between both parental strains is indicated. Insertions upstream of the coding sequence are also indicated.

Gene ID	Annotation	Total SNPs	Syn-SNPs	Non- Syn SNPs	Remarks
Zt09_11_00185	***Zymoseptoria* melanin regulation 1 (*Zmr1*). Nucleic acid binding, Zn ion binding (*Cmr1* homolog)**	1	1	0	~30 kb insertion (1.8 kb upstream)
Zt09_11_00186	**1,3,8-trihydroxynaphthalene reductase (*Thr1*)**	1	1	0	
Zt09_11_00187	No conserved domain	2	1	1	
Zt09_11_00188	Unknown	0	0	0	
Zt09_11_00189	Protein involved in establishing cohesion between sister chromatids during DNA replication	2		2	
Zt09_11_00190	Ureidoglycolate hydrolase *(Udh)*	2	2	0	13 bp deletion in promoter region, (215 bp upstream)
