# Supplementary material for: Transposable element insertions shape gene regulation and melanin production in a fungal pathogen of wheat
Source: BMC Biol. 2018 Jul 16;16:78. doi: 10.1186/s12915-018-0543-2 (PMC6047131; doi:10.1186/s12915-018-0543-2)
Supplement: Supplementary file 4 — Zmr1 expression levels are lower in 3D1 than in 3D7 at 7 days post inoculation. Mean and standard errors (se) of the relative quantification (RQ, fold change in expression level of Zmr1 with respect to the Zmr1 expression levels of the strain 3D1 at 7 days post inoculation, dpi) of the expression of Zmr1 in 3D1 and 3D7 at 7 and 9 dpi. The experiment was performed three times with similar results. (PDF 31 kb) [file 12915_2018_543_MOESM4_ESM.pdf]

Additional file 4. *Zmr1* expression levels are lower in 3D1 than in 3D7 at 7 days post inoculation. Mean and standard errors (se) of the relative quantification (RQ, fold change in expression level of *Zmr1* with respect to the *Zmr1* expression levels of the strain 3D1 at 7 days post inoculation, dpi) of the expression of *Zmr1* in 3D1 and 3D7 at 7 and 9 dpi. The experiment was performed three times with similar results.

Strains	dpi	Mean RQ	se
3D1	7	1.00	0.37
3D7	7	14.66	0.52
3D1	9	3.95	0.41
3D7	9	7.78	0.44
