# Supplementary figures and images for: Transposable element insertions shape gene regulation and melanin production in a fungal pathogen of wheat
Source: BMC Biol. 2018 Jul 16;16:78. doi: 10.1186/s12915-018-0543-2 (PMC6047131; doi:10.1186/s12915-018-0543-2)

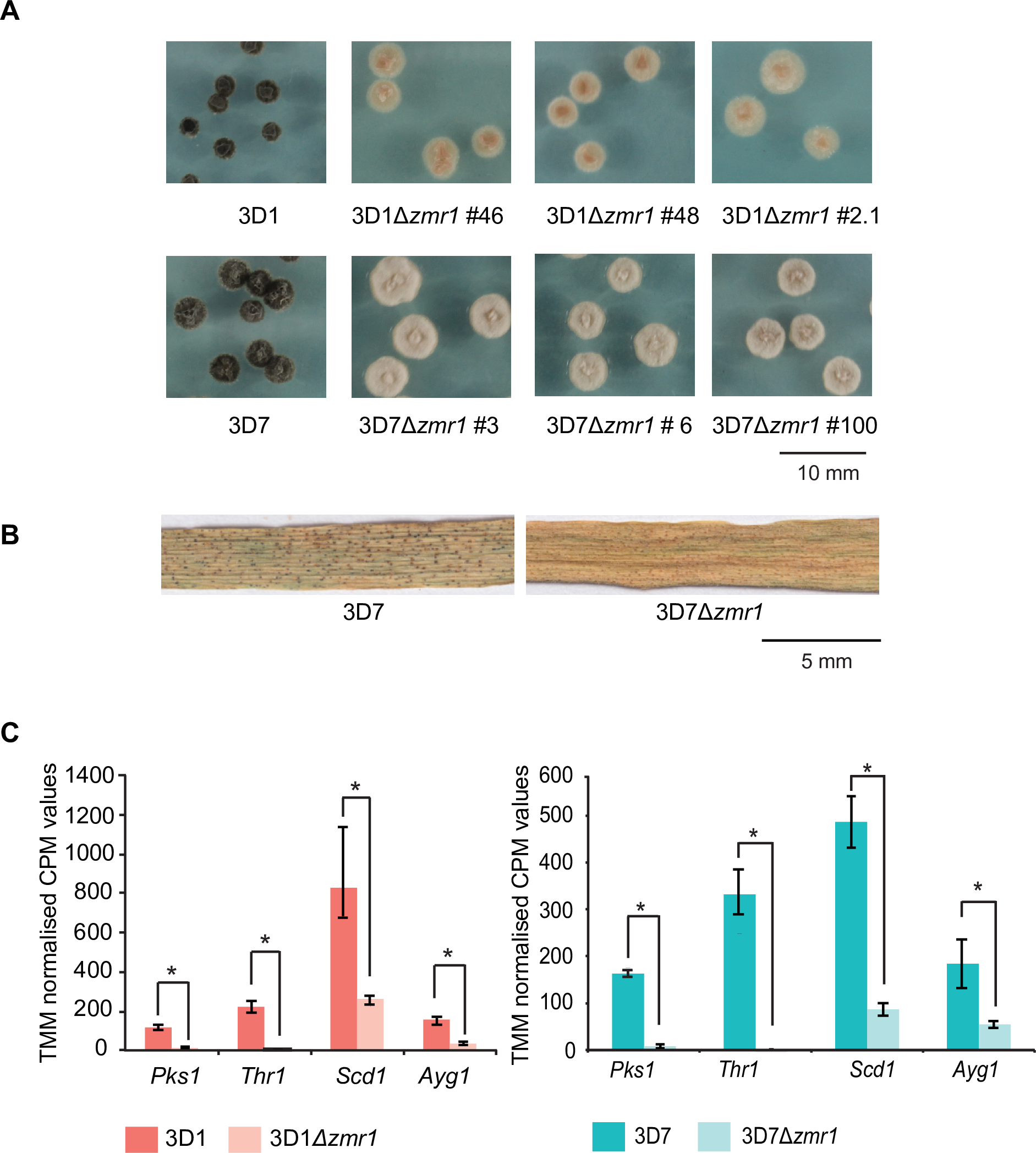

Supplement: Supplementary file 5 — Zmr1 regulates melanin biosynthesis in Z. tritici. (A) Three independent Zmr1 disruptant mutants in the 3D1 (#46, #48, and #2.1) and in the 3D7 (#3, #6, and #100) backgrounds lack melanin. Pictures of 10-day-old wild-type 3D7 and 3D7Δzmr1 colonies. (B) Melanized and albino pycnidia of 3D7 and 3D7Δzmr1, respectively, on wheat leaves of the cultivar Drifter, 21 dpi. (C) Expression values of the genes in the DHN melanin biosynthesis pathway (Pks1 = polyketide synthase 1; Thr1 = 1,3,8-trihydroxynaphthalene reductase; Zmr1 = Zymoseptoria melanin regulation 1; Ayg1 = Homolog of Aspergillus yellowish green) for the wild-type and Δzmr1 in the 3D1 and 3D7 backgrounds, respectively. Mean of TMM (trimmed mean of M values) normalized log2 CPM (counts per million mapped reads) values of three independent replicates with their standard deviation are plotted. Asterisks indicate statistical differences between the wild-type and the mutant (p value ≤ 0.05, FDR ≤ 0.05). (TIF 1614 kb) [file 12915_2018_543_MOESM5_ESM.tif]

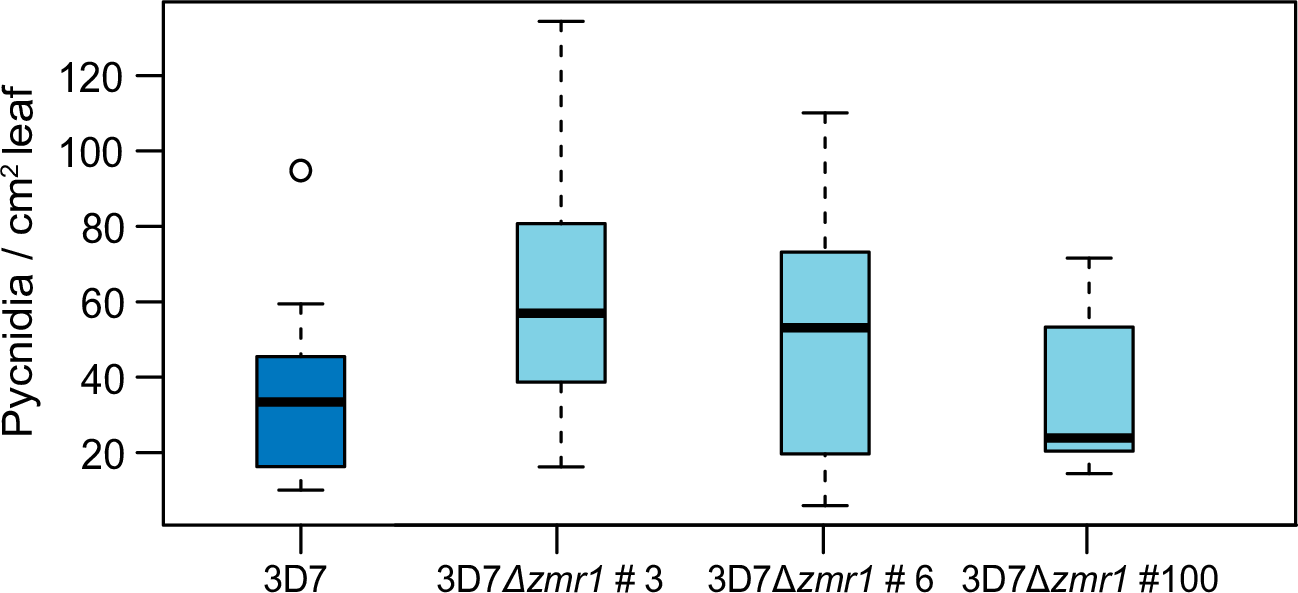

Supplement: Supplementary file 11 — Melanin is not essential for virulence. All three 3D7Δzmr1 mutants were equally virulent on the winter wheat variety Drifter, compared to the wild-type 3D7 as indicated by pycnidia/cm2 leaf 21 days post infection (Turkey’s HSD test, p value ≤ 0.05). Mean and standard error of the mean of 12 independent leaves are shown. The experiment was performed three times for line #6 with similar results. (TIF 86 kb) [file 12915_2018_543_MOESM11_ESM.tif]

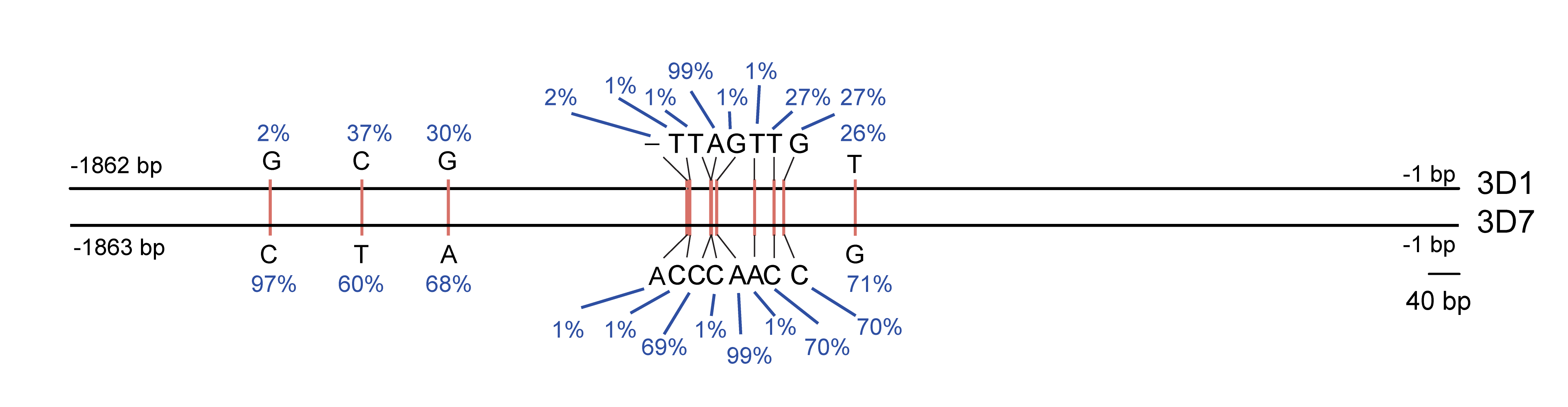

Supplement: Supplementary file 15 — SNPs in Zmr1 regulatory sequences. Alignment of the nucleotide sequences 1862 bp upstream of the coding sequence of Zmr1 in 3D1 and 3D7. Vertical pink bars indicate the 12 SNPs in the promoter region. Frequency (in percentage) of the 3D1 and 3D7 variants in the analyzed 132 strains of Z. tritici are indicated. (TIF 16001 kb) [file 12915_2018_543_MOESM15_ESM.tif]

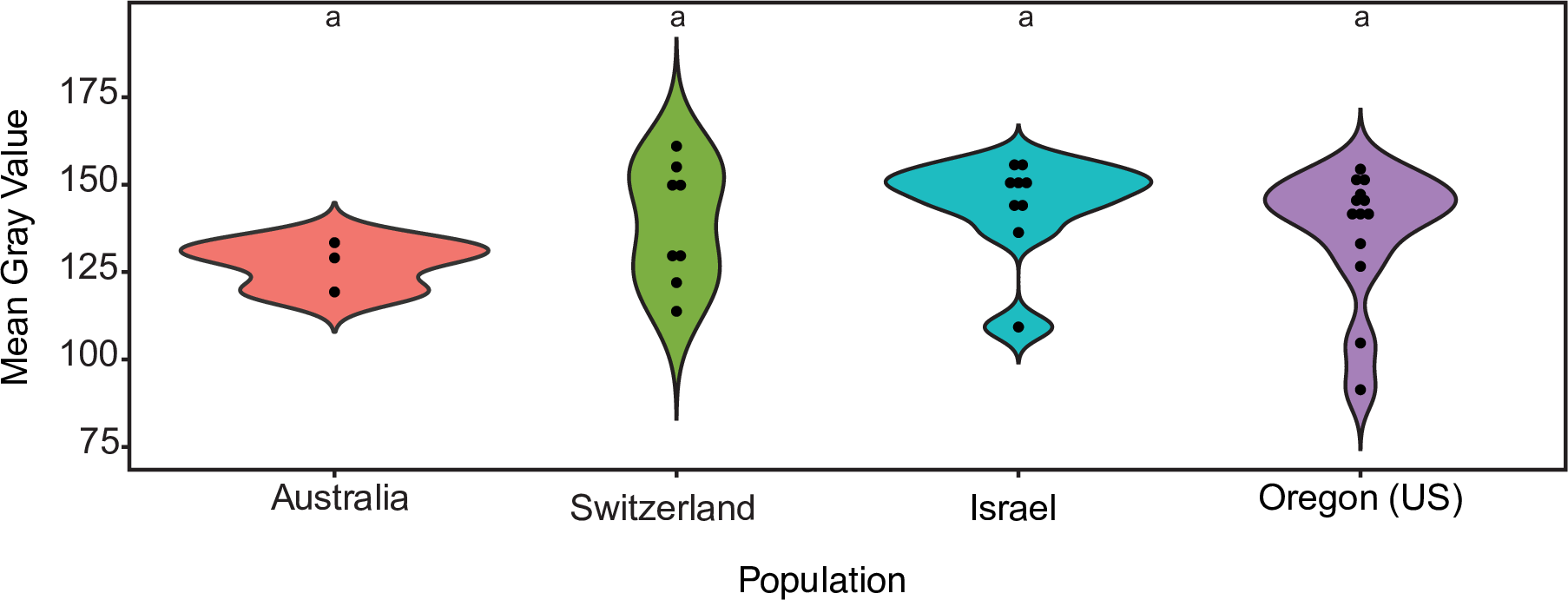

Supplement: Supplementary file 16 — No significant difference in the distribution of mean gray values of different Z. tritici strains belonging to four different populations. Distribution of mean gray values of different Z. tritici strains from four different populations across the world (3 strains from Australia, 8 from Switzerland, 9 from Israel, and 13 from the USA). The colors of the violin plot indicate different populations. Black dots represent individual data points, which correspond to the mean gray values of each Z. tritici strain at 7 days post inoculation. At least 100 colonies grown on five different plates were evaluated. The experiment was performed three times with similar results. No statistically significant differences were observed between the populations (Kruskal-Wallis test, p value ≤ 0.05). (TIF 172 kb) [file 12915_2018_543_MOESM16_ESM.tif]

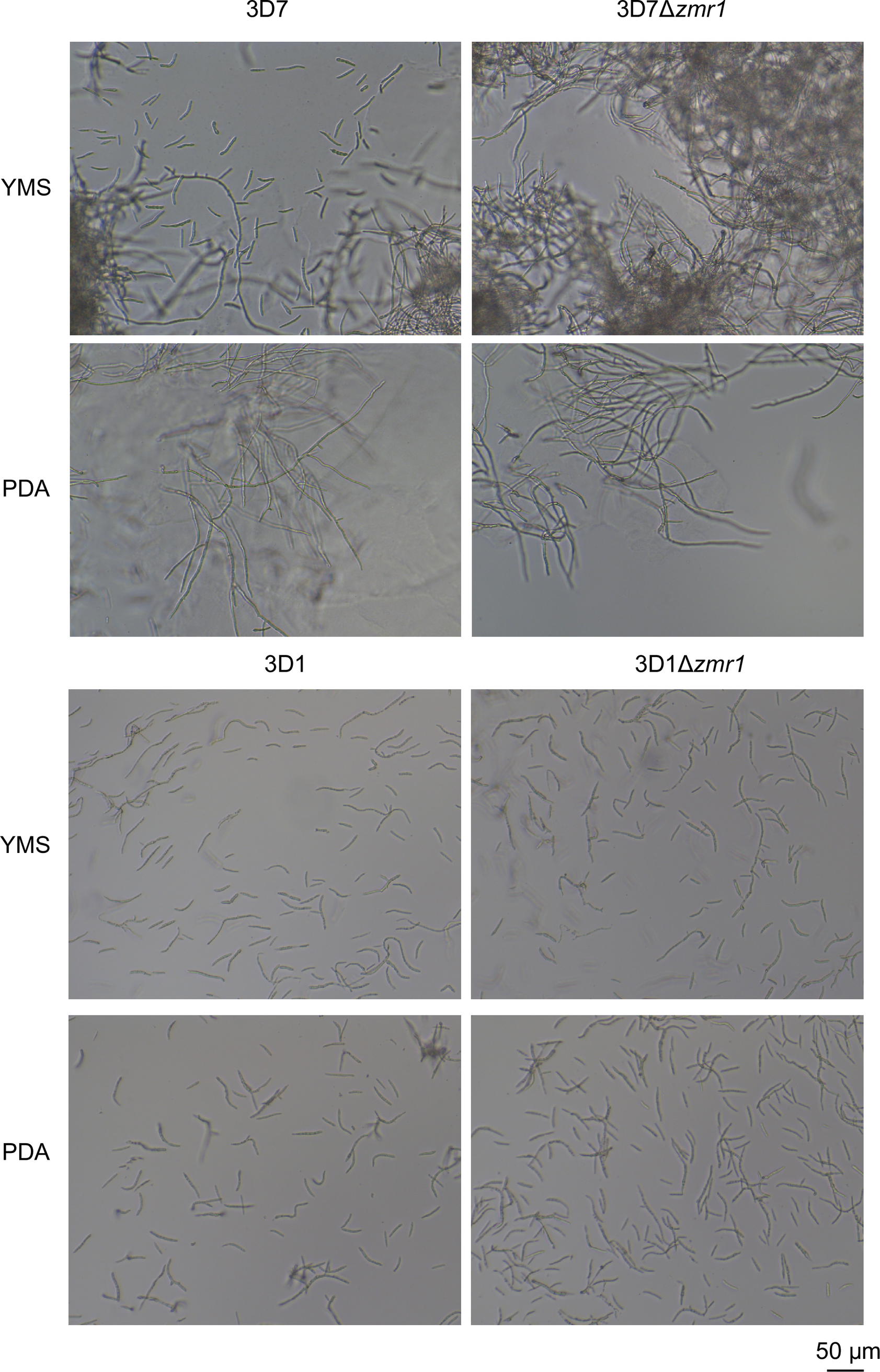

Supplement: Supplementary file 18 — Altered growth morphology of 3D7Δzmr1 mutants on YMS but not in PDA. Morphology of 3D1, 3D7, and the mutants in Zmr1 grown on YMS and PDA at 7 days post inoculation. (TIF 6379 kb) [file 12915_2018_543_MOESM18_ESM.tif]

**A**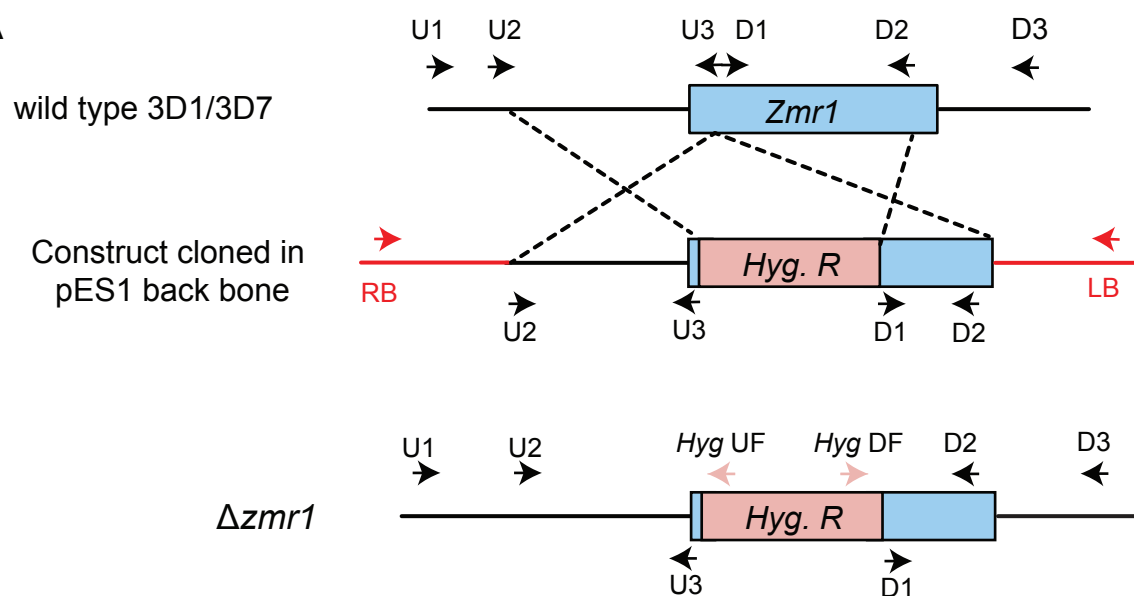**B**

wild type 3D1

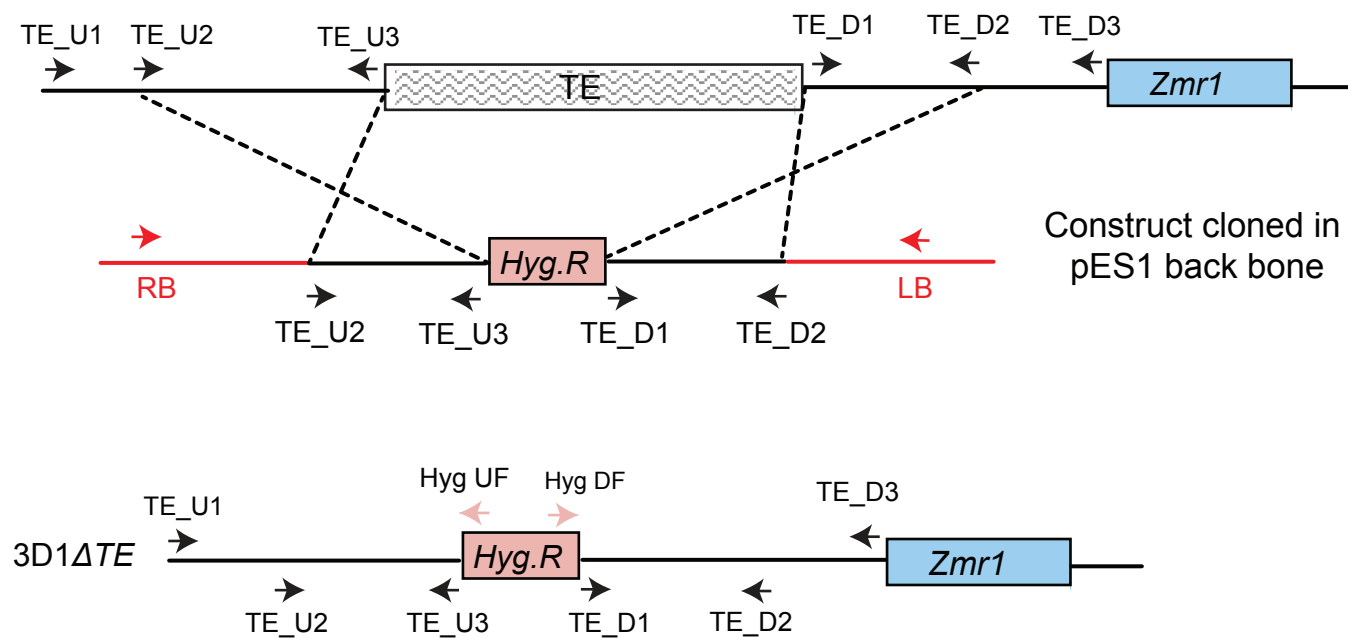

C

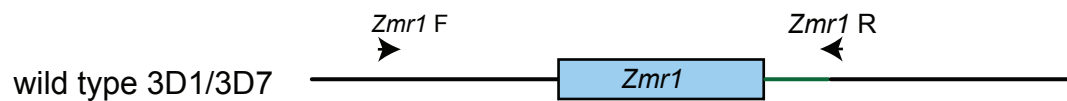

*Zmr1* + Geneticin resistant cassette  
cloned in pES1 back bone

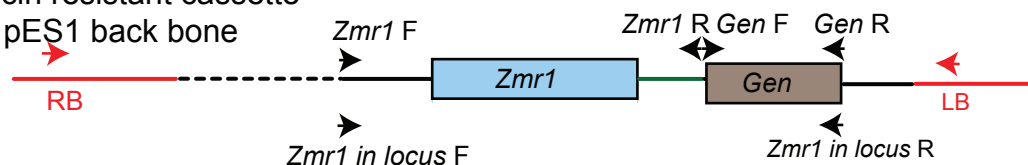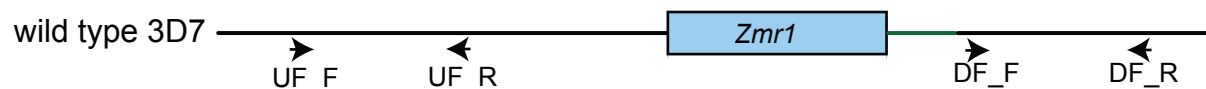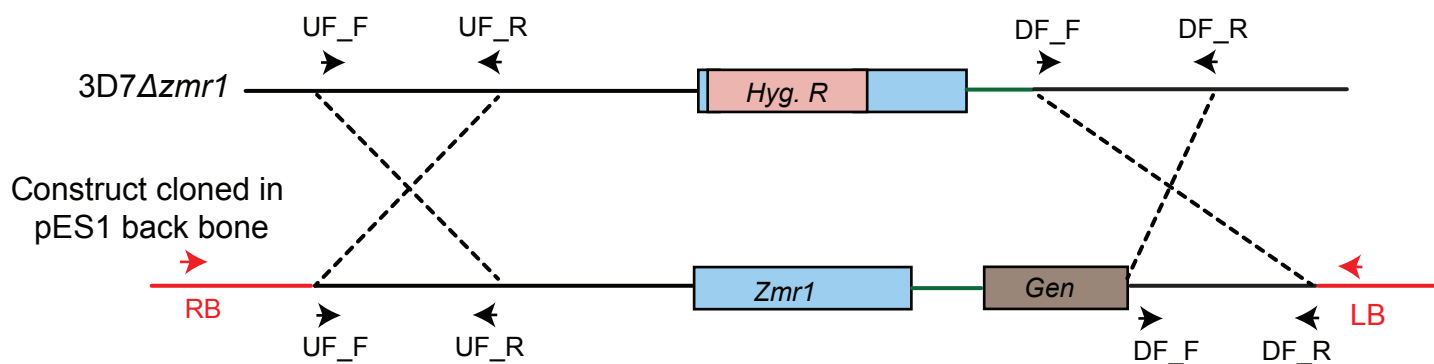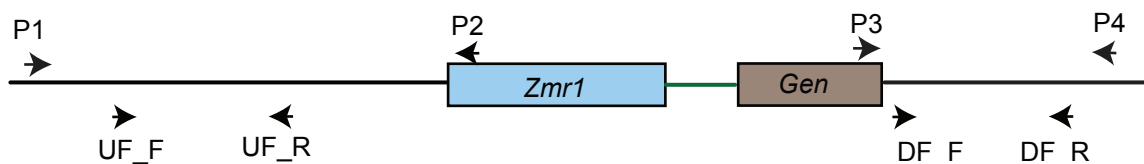

3D1 $\Delta$ *zmr1* + *Zmr1* (in locus)

Supplement: Supplementary file 19 — Generation of mutants (A) Schematic diagram showing the location of primers used for generating Zmr1 disruptant mutants (U2 + U3, D1 + D2) and the primers used for screening the transformants (U1 + Hyg UF, Hyg DF + D3). (B) Schematic diagram showing the location of primers used for generating the transposable element (TE) deletion mutants in 3D1 background (ΔTE; TE_U2 + TE_U3, TE_D1 + TE_D2) and the primers used for screening the transformants (TE_U1 + Hyg UF, Hyg DF + TE_D3). (C) Diagrams showing the location of primers used for generating the transformants expressing Zmr1 gene in locus in the 3D7Δzmr1 background. Amplification of Zmr1 gene from genomic DNA of 3D1 and 3D7 strains was performed using Zmr1 F and Zmr1 R primers; the geneticin resistance cassette (Gen) was amplified from pCGEN with Gen F and Gen R. Both amplicons were fused to the pES1 backbone to generate an intermediate construct, which was used to amplify Zmr1 and the geneticin resistance cassette using Zmr1 in locus F + Zmr1 in locus R. Up-flanking and down-flanking regions of the insertion site were amplified from 3D7 genomic DNA using the primers UF_F + UF_R and DF_F + DF_R. Primers used for screening the transformants (P1 + P2, P3 + P4) are also shown. LB and RB indicates the left and right border of the binary vector pES1. Hyg.R = hygromycin resistance cassette. (PDF 139 kb) [file 12915_2018_543_MOESM19_ESM.pdf]
