# Supplementary material for: Transposable element insertions shape gene regulation and melanin production in a fungal pathogen of wheat
Source: BMC Biol. 2018 Jul 16;16:78. doi: 10.1186/s12915-018-0543-2 (PMC6047131; doi:10.1186/s12915-018-0543-2)
Supplement: Supplementary file 6 — Reduced expression of genes in the Pks1 cluster in Δzmr1 mutants. Mean and standard error (se) of CPM (counts per million mapped reads) values of genes significantly downregulated (false discovery rates, FDR ≤ 0.05) in both Δzmr1 mutants compared to the wild-type strains 3D1 and 3D7. Means and standard errors of the mean of three independent replicates are indicated. Genes previously shown to be involved in melanin biosynthesis are shown in bold. (PDF 423 kb) [file 12915_2018_543_MOESM6_ESM.pdf]

Additional file 6. Reduced expression of genes in the *Pks1* cluster in *Δzmr1* mutants. Mean and standard error (se) of CPM (counts per million mapped reads) values of genes significantly down regulated (false discovery rates, FDR ≤ 0.05) in both *Δzmr1* mutants compared to the wild type strains 3D1 and 3D7. Means and standard errors of the mean three independent replicates are indicated. Genes previously shown to be involved in melanin biosynthesis are shown in bold.

Gene name	Annotation	3D1		3D1*Δzmr1*#48		3D7		3D7*Δzmr1*#6	
		Mean CPM	se	Mean CPM	se	Mean CPM	se	Mean CPM	se
Zt09_11_00186	**1,3,8-trihydroxynaphthalene reductase (*Thr1*)**	221.68	30.49	0.21	0.17	332.34	48.30	0.72	0.22
Zt09_4_00395	Similar to cytochrome p450	460.50	30.42	11.28	1.13	574.91	31.95	6.42	0.35
Zt09_2_00626	Hypothetical protein	243.46	70.04	19.34	0.55	61.05	4.35	20.08	2.46
Zt09_11_00184	**Polyketide synthase 1 (*Pks1*)**	117.12	13.72	12.55	3.22	162.81	6.79	7.82	2.46
Zt09_2_00074	Predicted protein	25.32	6.68	4.49	1.22	301.24	23.90	8.86	7.70
Zt09_3_00872	***Aspergillus* yellowish green homolog (*Ayg1*)**	156.39	18.63	34.11	6.83	184.04	52.82	54.39	8.24
Zt09_4_00393	Hypothetical protein	212.18	64.28	46.83	10.71	298.59	34.47	31.16	7.64
Zt09_1_00117	Hypothetical protein	43.81	13.52	9.92	0.86	150.38	34.73	8.58	1.35
Zt09_4_00394	Salicylate hydroxylase	216.33	35.00	48.75	7.50	230.78	31.04	43.61	3.75
Zt09_3_00063	Glycoside hydrolase family 18 protein	9.80	0.55	2.44	0.23	64.04	7.91	1.68	0.26
Zt09_11_00185	***Zymoseptoria* melanin regulation 1 (*Zmr1*)**	80.07	6.40	10.50	1.78	120.88	18.69	31.50	2.82
Zt09_1_00074	Hypothetical protein	25.32	6.68	4.49	1.22	301.24	23.90	8.86	7.70
Zt09_1_00268	**Scytalone dehydratase (*Scd1*)**	827.35	235.93	261.77	23.09	487.02	31.77	86.58	15.07
