# Supplementary material for: Transposable element insertions shape gene regulation and melanin production in a fungal pathogen of wheat
Source: BMC Biol. 2018 Jul 16;16:78. doi: 10.1186/s12915-018-0543-2 (PMC6047131; doi:10.1186/s12915-018-0543-2)
Supplement: Supplementary file 7 — Sequence variation in Zmr1 promoter contributes to differences in melanin accumulation. Means and standard errors of gray values (0 = black, 255 = white) of 3D1, 3D7, 3D7Δzmr1, 3D7Δzmr1 + Zmr13D1, and 3D7Δzmr1 + Zmr13D7, 7 days post inoculation (dpi) based on at least 20 colonies. Asterisks (*) and pluses (+) indicate significant differences in mean gray values of each strain with respect to the mean gray value of 3D7 and 3D7Δzmr1, respectively (Kruskal-Wallis, p values ≤ 0.05). The experiment was performed twice with similar results. NA = not applicable. (PDF 335 kb) [file 12915_2018_543_MOESM7_ESM.pdf]

Additional file 7. **Sequence variation in *Zmr1* promoter contributes to differences in melanin accumulation**. Means and standard errors of gray values (0 = black, 255 = white) of 3D1, 3D7, 3D7*Δzmr1*, 3D7*Δzmr1* + $Zmr1_{3D1}$ and 3D7*Δzmr1*+$Zmr1_{3D7}$, 7 days post inoculation (dpi) based on at least 20 colonies. Asterisks (*) and pluses (+) indicate significant differences in mean gray values of each strain with respect to the mean gray value of 3D7 and 3D7Δ*zmr1*, respectively (Kruskal-Wallis, p-values ≤ 0.05). The experiment was performed twice with similar results. NA = not applicable.

Strain	Allele of *Zmr1*	Mean gray value	Number of colonies analyzed (n)	Standard error
3D1	3D1	154 $^{*,+}$	70	0.7
3D7	3D7	109 $^{+}$	24	1.5
3D7Δ*zmr1*	NA	174 *	20	1.0
3D7Δ*zmr*1+ $Zmr1_{3D1}$ #4	3D1	120 $^{*,+}$	26	1.3
3D7Δ*zmr1* +$Zmr1_{3D1}$ #11	3D1	125 $^{*,+}$	93	0.9
3D7Δ*zmr1*+ $Zmr1_{3D1}$ #23	3D1	127 $^{*,+}$	25	2.3
3D7Δ*zmr1*+$Zmr1_{3D7}$ #12	3D7	114 $^{+}$	21	2.6
3D7Δ*zmr1*+$Zmr1_{3D7}$ # 14	3D7	112 $^{+}$	22	3.0
3D7Δ*zmr1*+$Zmr1_{3D7}$ # 72	3D7	100 $^{+}$	35	1.4
