# Supplementary material for: Transposable element insertions shape gene regulation and melanin production in a fungal pathogen of wheat
Source: BMC Biol. 2018 Jul 16;16:78. doi: 10.1186/s12915-018-0543-2 (PMC6047131; doi:10.1186/s12915-018-0543-2)
Supplement: Supplementary file 8 — The transposable element insertion upstream of Zmr1 in 3D1 downregulates Zmr1 expression. Mean gray values (0 = black, 255 = white) based on at least 35 colonies of the wild-types 3D1 and 3D7, three independent TE deletion mutants in the 3D1 background (3D1 ΔTE #93, #131, #239) and the two ectopic controls (3D1+ Hyg #3 and #6), 7 days post inoculation. Asterisks (*) indicate that the strains are significantly darker than the wild-type 3D1 (Kruskal-Wallis, p value ≤ 0.05). The experiment was performed three times with 3D1ΔTE #93 and twice with #131 and #239. (PDF 330 kb) [file 12915_2018_543_MOESM8_ESM.pdf]

**Additional file 8. The transposable element insertion upstream of *Zmr1* in 3D1 down-regulates *Zmr1* expression.** Mean gray values (0 = black, 255 = white) based on at least 35 colonies of the wild types 3D1 and 3D7, three independent TE deletion mutants in the 3D1 background (3D1 *ΔTE* #93, #131, #239) and the two ectopic controls (3D1+ Hyg #3 and #6), 7 days post inoculation. Asterisks (*) indicate that the strains are significantly darker than the wild type 3D1 (Kruskal-Wallis, p-value ≤ 0.05). The experiment was performed three times.

| Strain | Mean Gray Value | Number of colonies analyzed (n) | Standard error |
|---|---|---|---|
| 3D1 | 143 | 72 | 1.7 |
| 3D7 | 97 * | 36 | 1.5 |
| 3D1 + Hyg #3 | 160 | 132 | 1.3 |
| 3D1 + Hyg #6 | 157 | 83 | 0.5 |
| 3D1Δ*TE* #93 | 95 * | 122 | 1.4 |
| 3D1Δ*TE* #131 | 127 * | 65 | 1.9 |
| 3D1Δ*TE* #239 | 122 * | 54 | 1.5 |
