# Supplementary material for: Transposable element insertions shape gene regulation and melanin production in a fungal pathogen of wheat
Source: BMC Biol. 2018 Jul 16;16:78. doi: 10.1186/s12915-018-0543-2 (PMC6047131; doi:10.1186/s12915-018-0543-2)
Supplement: Supplementary file 9 — Non-melanized mutants grow faster than the wild-types. Radial growth rates (slope of the curves) of wild-type strains and melanin-deficient mutants (3D1Δzmr1 and 3D7Δzmr1) were obtained by plotting radial size (mm) of the colonies over time. The fit of the radial growth curve estimated using a linear model was evaluated using Pearson’s correlation coefficient (r2 value). Asterisks (*) indicate significant differences in growth rate (slope) between the wild-type and the mutant according to ANCOVA analysis (p values ≤ 0.05). The experiment was performed three times with similar results. (PDF 332 kb) [file 12915_2018_543_MOESM9_ESM.pdf]

Additional file 9. Non-melanized mutants grow faster than the wild types. Radial growth rates (slope of the curves) of wild type strains and melanin deficient mutants (3D1*Δzmr1* and 3D7*Δzmr1*) were obtained by plotting radial size (mm) of the colonies over time. The fit of the radial growth curve estimated using a linear model was evaluated using Pearson's correlation coefficient (r^2 value). Asterisks (*) indicate significant differences in growth rate (slope) between the wild type and the mutant according to ANCOVA analysis (p-values ≤ 0.05). The experiment was performed three times with similar results.

**Strains**	**r^2 values**	**Radial growth rate (Slope)**	**Number of colonies analyzed (n)**	**ANCOVA p-value**
3D1	0.98	0.14	64	
3D1*Δzmr1* #46	0.93	0.21	112	5.54e-04*
3D1*Δzmr1* #48	1	0.36	35	2.75e-08*
3D1*Δzmr1* #2.1	0.98	0.29	57	9.24e-07*
3D7	0.99	0.21	74	
3D7*Δzmr1* #3	0.91	0.29	61	4.7e-06*
3D7*Δzmr1* #6	1	0.32	68	3.26e-07*
3D7*Δzmr1* #100	0.91	0.3	53	6.48e-06*
