# Supplementary material for: Transposable element insertions shape gene regulation and melanin production in a fungal pathogen of wheat
Source: BMC Biol. 2018 Jul 16;16:78. doi: 10.1186/s12915-018-0543-2 (PMC6047131; doi:10.1186/s12915-018-0543-2)
Supplement: Supplementary file 10 — Colonies of non-melanized mutants are bigger than those of the corresponding wild-types. Means and standard errors of the radial size (mm) based on at least 20 colonies at different days post inoculation (dpi). The experiment was performed three times with similar results. (PDF 65 kb) [file 12915_2018_543_MOESM10_ESM.pdf]

**Additional file 10. Colonies of non-melanized mutants are bigger than those of the corresponding wild types.** Means and standard errors of the radial size (mm) based on at least 20 colonies at different days post inoculation (dpi). The experiment was performed three times with similar results.

| dpi | Strain | Radius (mm) | Standard error | dpi | Strain | Radius (mm) | Standard error |
|---|---|---|---|---|---|---|---|
| 8 | 3D1 | 0.91 | 0.0 | 8 | 3D7 | 1.11 | 0.0 |
| 9 | 3D1 | 1.10 | 0.1 | 9 | 3D7 | 1.39 | 0.0 |
| 10 | 3D1 | 1.25 | 0.1 | 10 | 3D7 | 1.60 | 0.1 |
| 11 | 3D1 | 1.35 | 0.1 | 11 | 3D7 | 1.78 | 0.1 |
| 12 | 3D1 | 1.46 | 0.1 | 12 | 3D7 | 1.97 | 0.1 |
| 8 | 3D1*Δzmr1* #46 | 1.07 | 0.0 | 8 | 3D7*Δzmr1* #3 | 2.02 | 0.0 |
| 9 | 3D1*Δzmr 1* #46 | 1.39 | 0.1 | 9 | 3D7*Δzmr1* #3 | 2.54 | 0.1 |
| 10 | 3D1*Δzmr1* #46 | 1.61 | 0.1 | 10 | 3D7*Δzmr1* #3 | 2.87 | 0.1 |
| 11 | 3D1*Δzmr1* #46 | 1.61 | 0.1 | 11 | 3D7*Δzmr1* #3 | 2.84 | 0.1 |
| 12 | 3D1*Δzmr1* #46 | 2.00 | 0.1 | 12 | 3D7*Δzmr1* #3 | 3.31 | 0.1 |
| 8 | 3D1*Δzmr1* #48 | 1.18 | 0.0 | 8 | 3D7*Δzmr1* #6 | 1.72 | 0.0 |
| 9 | 3D1*Δzmr1* #48 | 1.59 | 0.0 | 9 | 3D7*Δzmr1* #6 | 2.20 | 0.1 |
| 10 | 3D1*Δzmr1* #48 | 1.95 | 0.1 | 10 | 3D7*Δzmr1* #6 | 2.44 | 0.1 |
| 11 | 3D1*Δzmr1* #48 | 2.29 | 0.1 | 11 | 3D7*Δzmr1* #6 | 2.74 | 0.1 |
| 12 | 3D1*Δzmr1* #48 | 2.62 | 0.1 | 12 | 3D7*Δzmr1* #6 | 3.04 | 0.2 |
| 8 | 3D1*Δzmr1* #2.1 | 1.26 | 0.0 | 8 | 3D7*Δzmr1* #100 | 1.92 | 0.0 |
| 9 | 3D1*Δzmr1* #2.1 | 1.66 | 0.1 | 9 | 3D7*Δzmr1* #100 | 2.47 | 0.0 |
| 10 | 3D1*Δzmr1* #2.1 | 1.98 | 0.1 | 10 | 3D7*Δzmr1* #100 | 2.84 | 0.1 |
| 11 | 3D1*Δzmr1* #2.1 | 2.22 | 0.1 | 11 | 3D7*Δzmr1* #100 | 2.84 | 0.1 |
| 12 | 3D1*Δzmr1* #2.1 | 2.44 | 0.1 | 12 | 3D7*Δzmr1* #100 | 3.21 | 0.3 |
