# Supplementary material for: Transposable element insertions shape gene regulation and melanin production in a fungal pathogen of wheat
Source: BMC Biol. 2018 Jul 16;16:78. doi: 10.1186/s12915-018-0543-2 (PMC6047131; doi:10.1186/s12915-018-0543-2)
Supplement: Supplementary file 12 — Melanin-deficient mutants are not impaired in virulence. Mean of percentage of leaf area covered by lesions (PLACL) and pycnidia/cm2 leaf caused by the wild-type 3D7 and 3D7Δzmr1 lines on the wheat cultivar Drifter, at 22 days post inoculation. Mean and standard error of the mean of 12 leaves are shown. The experiment was performed twice with the line 6 and 3D7 obtaining similar results. No statistical differences in PLACL and pycnidia/cm2 leaf were detected between 3D7 and 3D7∆zmr1 according to Tukey’s HSD (honest significant difference) test (p values ≤ 0.05). (PDF 223 kb) [file 12915_2018_543_MOESM12_ESM.pdf]

Additional file 12. Melanin deficient mutants are not impaired in virulence. Mean of percentage of leaf area covered by lesions (PLACL) and pycnidia/cm^2 leaf caused by the wildtype 3D7 and 3D7Δ*zmr1* lines on the wheat cultivar Drifter, at 22 days post inoculation. Mean and standard error of the mean of 12 leaves are shown. The experiment was performed twice with the line 6 and 3D7 obtaining similar results. No statistical differences in PLACL and pycnidia/cm^2 leaf were detected between 3D7 and 3D7Δ*zmr1* according to Tukey's HSD (honest significant difference) test (p-values ≤ 0.05).

Strain	PLACL	Standard error	pycnidia/cm^2 leaf	Standard error
3D7	53	2.8	35	7.0
3D7*Δzmr1*#3	61	5.0	61	9.8
3D7*Δzmr1*#6	40	8.0	53	9.9
3D7*Δzmr1*#100	46	4.2	35	5.8
