# Supplementary material for: Transposable element insertions shape gene regulation and melanin production in a fungal pathogen of wheat
Source: BMC Biol. 2018 Jul 16;16:78. doi: 10.1186/s12915-018-0543-2 (PMC6047131; doi:10.1186/s12915-018-0543-2)
Supplement: Supplementary file 13 — Melanin protects Z. tritici against SDHI fungicides. Reduction in growth by fungicides (% growth decrease) of the wild-type 3D7 and 3D7Δzmr1. Strains were grown on Whatman filter paper placed on PDA for 5 days and later transferred to PDA plates supplemented with the fungicide bixafen or propiconazole. Asterisks (*) indicate that the percentage in decrease in growth of the mutant strain, in the presence of the fungicide, is significantly different from the wild-type 3D7 (Kruskal-Wallis, p value ≤ 0.05). Mean and standard error of differential radial size of at least 25 colonies grown on three independent plates are presented. The experiment was performed twice with similar results. (PDF 122 kb) [file 12915_2018_543_MOESM13_ESM.pdf]

Additional file 13. **Melanin protects *Z. tritici* against SDHI fungicides**. Reduction in growth by fungicides (% growth decrease) of the wild type 3D7 and 3D7*Δzmr1*. Strains were grown on Whatman filter paper placed on PDA for 5 days and later transferred to PDA plates supplemented with the fungicide bixafen or propiconazole. Asterisks (*) indicate that the percentage in decrease in growth of the mutant strain, in the presence of the fungicide, is significantly different from the wild type 3D7 (Kruskal-Wallis, p-value ≤ 0.05). Mean and standard error of differential radial size of at least 25 colonies grown on three independent plates are presented. The experiment was performed twice with similar results.

**Bixafen 0.75 ppm**			
**dpi**	**Strain**	**% Growth decrease**	**Standard error**
8	3D7	40	4.8
9	3D7	49	4.2
10	3D7	50	2.7
11	3D7	51	3.0
12	3D7	51	1.9
8	3D7Δ*zmr1* #6	42	1.9
9	3D7Δ*zmr1* #6	53	1.9
10	3D7Δ*zmr1* #6	54	1.6
11	3D7Δ*zmr1* #6	58*	1.1
12	3D7Δ*zmr1* #6	58*	1.7
**Propiconozole 0.75 ppm**			
**dpi**	**Strain**	**% Growth decrease**	**Standard error**
8	3D7	28	2.0
9	3D7	38	6.6
10	3D7	31	5.5
11	3D7	28	8.5
12	3D7	36	5.7
8	3D7Δ*zmr1* #6	27	1.9
9	3D7Δ*zmr1* #6	32	2.7
10	3D7Δ*zmr1* #6	32	2.0
11	3D7Δ*zmr1* #6	33	0.9
12	3D7Δ*zmr1* #6	33	1.8
