# Supplementary material for: Transposable element insertions shape gene regulation and melanin production in a fungal pathogen of wheat
Source: BMC Biol. 2018 Jul 16;16:78. doi: 10.1186/s12915-018-0543-2 (PMC6047131; doi:10.1186/s12915-018-0543-2)
Supplement: Supplementary file 17 — Melanization and Zmr1 expression levels in Z. tritici strains from around the world. Means and standard errors of the mean of gray values (0 = black, 255 = white) and of Zmr1 expression at 7 days post inoculation. n indicates the number of colonies analyzed. Gray values were measured in three independent experiments, and similar results were obtained. Expression analysis was performed twice and provided similar results. AUS: Australia, CH: Switzerland, ISY: Israel and OR: USA (Oregon), TE = transposable element, P = Present, A = Absent. (PDF 205 kb) [file 12915_2018_543_MOESM17_ESM.pdf]

Additional file 17. Melanization and *Zmr1* expression levels in *Z. tritici* strains from around the world. Means and standard errors of the mean of gray values (0 = black, 255 = white) and of *Zmr1* expression at 7 days post inoculation. n indicates the number of colonies analyzed. Gray values were measured in three independent experiments, and similar results were obtained. Expression analysis was performed twice and provided similar results. AUS: Australia, CH: Switzerland, ISY: Israel and OR: USA (Oregon), TE = transposable element, P = Present, A = Absent.

Strains	Population	Mean Gray value	n	Standard error (gray value)	TE present/ absent	*Zmr1* expression levels relative to 3D1	Standard error (expression)
AUS_1D4	AUS	133	140	0.9	P	1.97	0.52
AUS_1F3	AUS	119	130	4.0	A	934.71	0.03
AUS_1E5	AUS	129	172	9.8	A	60.5	0.03
CH.ST99CH_3D1	CH	161	43	0.6	P	1.00	0.08
CH.ST99CH_3A2	CH	155	122	0.4	P	2.36	0.05
CH.ST99CH_3G6	CH	151	109	0.4	A	1.73	0.01
CH.ST99CH_3A6	CH	130	122	1.3	A	6.45	0.03
CH.ST99CH_3D8	CH	122	112	2.9	A	17.54	0.01
CH.ST99CH_1E4	CH	130	70	1.3	A	11.19	0.01
CH.ST99CH_1A5	CH	149	21	0.6	A	0.49	0.45
CH.ST99CH_3D7	CH	114	25	4.6	A	4.41	0.01
ISY_Ar_16h	ISY	144	128	0.7	P	2.67	0.02
ISY_Ar_5a	ISY	150	71	0.4	P	0.72	0.01
ISY_Ar_21a	ISY	151	137	0.4	P	1.36	<0.01
ISY_Ar_12e	ISY	157	211	0.2	P	1.33	0.01
ISY_Ar 11i	ISY	155	356	0.5	A	2.47	0.03
ISY_Ar 22f	ISY	150	384	0.2	A	1.54	<0.01

ISY_Ar 1c	ISY	136	505	0.4	A	8.4	0.03
ISY_Ar 19e	ISY	145	262	0.4	A	5.93	0.01
ISY_Ar 15a	ISY	109	590	0.4	A	33.74	0.01
ORE.R_a12_3B_8	OR	146	236	0.5	A	141.52	0.01
ORE.R_a12 3B.2	OR	142	90	1.4	A	68.67	0.01
ORE.R_a12 4A.1	OR	133	47	2.2	A	1753.28	0.10
ORE.S_a15_4A_10	OR	147	274	5.7	P	366.45	<0.01
ORE.S_a15_4A_15	OR	145	36	2.2	P	27.19	0.02
ORE.S_a15_3B_5	OR	152	58	0.5	P	29.47	0.01
ORE.S_a15 3B.18	OR	151	38	0.7	A	22.53	0.04
ORE.S_a15 2A.13	OR	91	91	1.4	A	443.96	0.05
ORE.S_a15 2A.8	OR	142	176	0.6	A	258.45	0.01
ORE.S_a15_ 2A.20	OR	105	202	0.5	A	3537.87	<0.01
ORE.S_a15 4A.2	OR	141	178	0.7	A	128.49	0.01
ORE.S_a15 3B.19	OR	127	160	1.4	A	272.79	<0.01
ORE.S_a15 4A_3	OR	154	83	0.9	P	33.84	0.01
