# Supplementary material for: Transposable element insertions shape gene regulation and melanin production in a fungal pathogen of wheat
Source: BMC Biol. 2018 Jul 16;16:78. doi: 10.1186/s12915-018-0543-2 (PMC6047131; doi:10.1186/s12915-018-0543-2)
Supplement: Supplementary file 20 — List of primers used in the study, their sequence, and their purpose. (PDF 287 kb) [file 12915_2018_543_MOESM20_ESM.pdf]

Additional file 20. List of primers used in the study, their sequence and their purpose

Primer Name	Name in the figure	Primer Sequence (5'-3')	Purpose
***zmr1* disruption mutants (S5 Fig)**			
u1_Zmr1	U1	TTCGAGTGTAAGCGTCATGC	Verification of the integration (with Hyg_pES6_seq_UF)
u2_Zmr1_Inf	U2	GATTACGAATTCTTAATTAAGAATTCAATG TGGACGATGGCCCA	Amplification of up-flanking region (with u3_Zmr1_Inf)
u3_Zmr1_inf	U3	ATCAAAGCATGTGCAGAAGACCATCTTCG C	Amplification of up-flanking region (with u2_Zmr1_Inf)
k1_Zmr1_inf	Not indicated	TGCACATGCTTTGATATTGAAGGAGCATT TTTTGGG	Amplification of Hygromycin resistance cassette (with k2_Zmr1_inf)
k2_Zmr1_inf	Not indicated	CCCGCAATGGCTAGCAGATCTCTATTCCT TTGC	Amplification of Hygromycin resistance cassette (with k2_Zmr1_inf)
d1_Zmr1_inf	D1	GCTAGCCATTGCGGGCAGTCATTCACT	Amplification of down-flanking region (with d2_Zmr1_inf)
d2_Zmr1_inf	D2	CCAAGCTTGCATGCCTGCAGGAATCATA GCGGGCATTTCATTGC	Amplification of down-flanking region (with d2_Zmr1_inf)
d3_Zmr1	D3	ACGGAATTGGCGTAGTTGAC	Verification of right Integration (with Hyg_pES6_seq_DF)
Hyg_pES6_ seq_UF	*Hyg* UF	GAACCATCTTGTCAAACGACAC	Verification of the integration (with u1_Zmr1)
Hyg_pES6_ seq_DF	*Hyg* DF	ACTGTCGGGCGTACACAAATC	Verification of the integration (with d3_Zmr1)
**TE_ knockout mutants (S6 Fig)**			
TE_KO_U1	TE_U1	Same as 3D7 insitu_U1	Verification of the integration (with Hyg_pES6_seq_UF)
TE_KO_U2	TE_U2	GATTACGAATTCTTAATTAACCAAGTGCG GAGAATATG	Amplification of up-flanking region (with TE_U3)

TE_KO_U3	TE_U3	TCAATATCTACGAGGTACTATATCCGCAC A	Amplification of up-flanking region (with TE_U2)
TE_KO_K1	TE_K1	TAGTACCTCGTAGATATTGAAGGAGCATT TTTTGGG	Amplification of Hygromycin resistance cassette (with TE_K2)
TE_KO_K2	TE_K2	GGAATGGAGGGCTAGCAGATCTCTATTC CTTTGC	Amplification of Hygromycin resistance cassette (with TE_K3)
TE_KO_D1	TE_D1	ATCTGCTAGCCCTCCATTCCACGTTTCG	Amplification of down-flanking region (with TE_D2)
TE_KO_D2	TE_D2	CCAAGCTTGCATGCCTGCAGGCCGCGAC AAGATTTGACTT	Amplification of down-flanking region (with TE_D3)
TE_KO_D3	TE_D3	Same as u3_Zmr1_inf	Verification of the integration (with Hyg_pES6_seq_DF)
***In locus* complementation of *Zmr1* in 3D7Δ*zmr1* (Fig 7)**			
Zmr1ect_F1	*Zmr1* F	GATTACGAATTCTTAATTAATCTCCATTCC ACGTTTCGC	Amplification of *Zmr1* gene (including promoter and terminator, with Zmr1ect_R1)
Zmr1ect_R1	*Zmr1* R	ACGATTCGAAGCCGCTACAGCGGGATGG GTGCT	Amplification of *Zmr1* gene (including promoter and terminator, with Zmr1ect_F1)
g418ect_F1	*Gen* F	GCGGCTTCGAATCGTGGC	Amplification of geneticin resistance cassette (with g418ect_R1)
g418ect_R1	Gen R	CCAAGCTTGCATGCCTGCAGGCCCTAGG TCGACGCCA	Amplification of geneticin resistance cassette (with g418ect_F1)
3D7_ insitu_U1	U1	GCTTACGTCGGAGCAGGTAC	Verification of the integration (with Zmr1_insitu_P2)
3D7_ insitu_U2	UF_F	GATTACGAATTCTTAATTAATATGTGTGGC TGGCTGAGAC	Amplification of up-flanking region (with 3D7_ insitu_U3)
3D7_ insitu_U3	UF_R	AAACGTGGAATGGAGTACGAGGTACTATA TCCGCACA	Amplification of up-flanking region (with 3D7_ insitu_U2)
3D7_ insitu_D1	DF_F	TGGCGTCGACCTAGGGTGCACATCCCTC CAACA	Amplification of down-flanking region (with 3D7_ insitu_D2)
3D7_ insitu_D2	DF_R	CCAAGCTTGCATGCCTGCAGGTGACGGT GATCTTCTTGTCG	Amplification of down-flanking region (with 3D7_ insitu_D1)

3D7_ insitu_D3	P4	TCTCGCCATTGGGAATGTAC	Verification of the integration (with Zmr1_insitu_P3)
Zmr1_F	*Zmr1* in locus F	same as Zmr1ect_F1	Amplification of *Zmr1*- geneticin resistance cassette (with Zmr1_R)
Zmr1_R	*Zmr1 in locus* R	same as g418ect_R1	Amplification of *Zmr1*- geneticin resistance cassette (with Zmr1_F)
Zmr1_insitu_ P2	P2	same as u3_Zmr1_inf	Verification of the integration (with 3D7_ insitu_U1)
Zmr1_insitu_ P3	P3	same as Zmr1ectgen_F	Verification of the integration (with 3D7_ insitu_D3)
**Primers used for qPCR**			
Zt18Sq_F	NA	CGCAGCAAATCCCACGG	qRT-PCR reference gene (with Zt18S_R)
Zt18Sq_R	NA	GCGCAGCTTCTTCCACTTTGAC	qRT-PCR reference gene (with Zt18S_F)
FL_TFIIIC1_ F	NA	TGCTCAGATTGTGCGAAGAC	qPCR reference gene (with FL_TFIIIC1_R)
FL_TFIIIC1_ R	NA	TCGTAGTCCGATACCATGAGG	qPCR reference gene (with FL_TFIIIC1_F)
GenR_q_F	NA	CTGTGCTCGACGTTGTCACT	qPCR for copy number detection (wit GenR_q_R)
GenR_q_R	NA	ATACTTTCTCGGCAGGAGCA	qPCR for copy number detection (with GenR_q_F)
HygR_q_F	NA	CGTCTGCTGCTCCATACA AG	qPCR for copy number detection (with HygR_q_R)
HygR_q_R	NA	CTCGATGAGCTGATGCTT TG	qPCR for copy number detection (with HygR_q_F)
Zmr1_SI1_F	NA	CCATATTCTCACACATACCAATG	qRT-PCR for expression analysis (with Zmr1_SI1_R)
Zmr1_SI1_R	NA	CGCATTTCGTCTTCGTCTTC	qRT-PCR for expression analysis (with Zmr1_SI1_F)
