# Supplementary material for: Transposable element insertions shape gene regulation and melanin production in a fungal pathogen of wheat
Source: BMC Biol. 2018 Jul 16;16:78. doi: 10.1186/s12915-018-0543-2 (PMC6047131; doi:10.1186/s12915-018-0543-2)
Supplement: Supplementary file 21 — Sample size and number of independent replicates performed in each experiment. In the experiments where gray value and radial growth were measured, n indicates the lowest number of colonies per strain analyzed in the experiment. In the experiments where expression levels of Zmr1 were measured, n indicates the number of technical replicates. (PDF 113 kb) [file 12915_2018_543_MOESM21_ESM.pdf]

Additional file 21 (Additional file 21.pdf) **Sample size and number of independent replicates performed in each experiment**. In the experiments where gray value and radial growth were measured, n indicates the lowest number of colonies per strain analyzed in the experiment. In the experiments where expression levels of *Zmr1* was measured, n indicates the number of technical replicates.

Figure	Description of the experiment	n	Number of independent experiments performed
1A	Gray value measurement of 3D1 and 3D7	60	3
3	Expression levels of *Zmr1* in 3D1 and 3D7	3	3
4	Gray value measurements of 3D7Δ*zmr1*+ Zmr1	20	2
6A	Gray value measurements of transposable element knockouts	35	3
2	Expression levels of *Zmr1* in transposable element knockouts	3	2
7A	Growth rate of 3D1 and 3D1Δ*zmr1*	35	3
7B	Growth rate of 3D7 and 3D7Δ*zmr1*	50	3
7C	Fungicide sensitivity of 3D7 and 3D7Δ*zmr1*	50	2
8B	Gray value measurements *Z. tritici* strains from 4 different populations	20	3
8C	Expression levels of *Zmr1* in *Z. tritici* strains from 4 different populations	3	2
Additional file 5C	RNA-seq analysis	-	3
Additional file 11	*In planta* virulence assay	12	1 (Performed twice with line 3D7Δzmr1 *#6*)
Additional file 14A	Gray value measurements of IPO323	50	2
